# Genomic and transcriptomic characterization of the human glioblastoma cell line AHOL1

**DOI:** 10.1590/1414-431X20209571

**Published:** 2021-01-15

**Authors:** W.A.S. Ferreira, C.K.N. Amorim, R.R. Burbano, R.A.R. Villacis, F.A. Marchi, T.S. Medina, M.M.C. de Lima, E.H.C. de Oliveira

**Affiliations:** 1 Instituto Evandro Chagas, Laboratório de Cultura de Tecidos e Citogenética, SAMAM, AnanindeuaPA Brasil Laboratório de Cultura de Tecidos e Citogenética, SAMAM, Instituto Evandro Chagas, Ananindeua, PA, Brasil; 2 Universidade Federal do Pará, Laboratório de Citogenética Humana, Instituto de Ciências Biológicas, BelémPA Brasil Laboratório de Citogenética Humana, Instituto de Ciências Biológicas, Universidade Federal do Pará, Belém, PA, Brasil; 3 Hospital Universitário João de Barros Barreto, Núcleo de Pesquisas em Oncologia, BelémPA Brasil Núcleo de Pesquisas em Oncologia, Hospital Universitário João de Barros Barreto, Belém, PA, Brasil; 4 Hospital Ophir Loyola, Laboratório de Biologia Molecular, BelémPA Brasil Laboratório de Biologia Molecular, Hospital Ophir Loyola, Belém, PA, Brasil; 5 Universidade de Brasília, Departamento de Genética e Morfologia, Instituto de Ciências Biológicas, BrasíliaDF Brasil Departamento de Genética e Morfologia, Instituto de Ciências Biológicas, Universidade de Brasília, Brasília, DF, Brasil; 6 A.C. Camargo Cancer Center, Centro Internacional de Pesquisa, São PauloSP Brasil Centro Internacional de Pesquisa, A.C. Camargo Cancer Center, São Paulo, SP, Brasil; 7 Universidade Federal do Pará, Instituto de Ciências Biológicas, Faculdade de Biomedicina, BelémPA Brasil Instituto de Ciências Biológicas, Faculdade de Biomedicina, Universidade Federal do Pará, Belém, PA, Brasil; 8 Universidade Federal do Pará, Instituto de Ciências Exatas e Naturais, Faculdade de Ciências Naturais, BelémPA Brasil Instituto de Ciências Exatas e Naturais, Faculdade de Ciências Naturais, Universidade Federal do Pará, Belém, PA, Brasil

**Keywords:** Array-comparative genomic hybridization, Gliomas, Transcriptomics, Brain tumors, Cell line, Glioblastoma

## Abstract

Cancer cell lines are widely used as *in vitro* models of tumorigenesis, facilitating fundamental discoveries in cancer biology and translational medicine. Currently, there are few options for glioblastoma (GBM) treatment and limited *in vitro* models with accurate genomic and transcriptomic characterization. Here, a detailed characterization of a new GBM cell line, namely AHOL1, was conducted in order to fully characterize its molecular composition based on its karyotype, copy number alteration (CNA), and transcriptome profiling, followed by the validation of key elements associated with GBM tumorigenesis. Large numbers of CNAs and differentially expressed genes (DEGs) were identified. CNAs were distributed throughout the genome, including gains at Xq11.1-q28, Xp22.33-p11.1, Xq21.1-q21.33, 4p15.1-p14, 8q23.2-q23.3 and losses at Yq11.21-q12, Yp11.31-p11.2, and 15q11.1-q11.2 positions. Nine druggable genes were identified, including *HCRTR2*, *ETV1*, *PTPRD*, *PRKX*, *STS*, *RPS6KA6*, *ZFY*, *USP9Y*, and *KDM5D*. By integrating DEGs and CNAs, we identified 57 overlapping genes enriched in fourteen pathways. Altered expression of several cancer-related candidates found in the DEGs-CNA dataset was confirmed by RT-qPCR. Taken together, this first comprehensive genomic and transcriptomic landscape of AHOL1 provides unique resources for further studies and identifies several druggable targets that may be useful for therapeutics and biologic and molecular investigation of GBM.

## Introduction

Glioblastomas (GBMs) are heterogeneous primary brain tumors that are likely originated from oligodendrocyte precursor cells, neural stem cells (NSCs), and NSC-derived astrocytes. They are the most lethal and common malignancy among all brain tumors, with an incidence rate of 3.21 cases per 100,000 individuals, median survival rate of 12-18 months, and higher predominance in males. GBMs are commonly diagnosed in elderly patients (median of 65 years), increasing with age, peaking at 75-84 years, and declining after 85 years (1).

According to the new classification for central nervous system (CNS) tumors proposed by the World Health Organization (WHO) in 2016, GBMs are classified as grade IV and included in diffuse astrocytic and oligodendroglial tumors. Based on the mutational pattern of the isocitrate dehydrogenase (IDH) gene, they are further classified as i) IDH-wildtype GBM (90% of cases), which frequently correspond to clinically defined primary GBM (or de novo GBM), arising predominately from the supratentorial region in patients with median age of ∼62 years at diagnosis and whose mean length of clinical history is 4 months; or ii) IDH-mutant-type GBM (10% of cases), which are defined as secondary GBM preferentially arising from the frontal region of younger patients (median age at diagnosis of ∼44 years) and whose prognosis is usually better than those with IDH-wildtype (1).

Currently, standard treatment for both GBM entities encompasses surgical resection followed by radiotherapy and chemotherapy (mainly using temozolomide - TMZ). However, these aggressive treatments are not effective in controlling the disease, thus indicating a high demand for new efficacious therapies to improve outcomes of patients with GBM (1).

*In vitro* cultures of GBM cell lines have been widely used as an important model for understanding GMB heterogeneity, drug sensitivity and resistance, evaluation of new therapeutic approaches, and to search for novel biomarkers. The Human Glioblastoma Cell Culture (HGCC) biobank has assembled a panel of 53 cell lines derived from surgical samples of GBM patients. However, there is a limited number of GBM cell lines deposited in HGCC or other biobanks (Broad-Novartis Cancer Cell Line Encyclopedia and American Type Culture Collection), given the heterogeneity of each molecular subtype of GBM (2). Thus, new, well-characterized cell lines that resemble these different molecular subtypes of GBM are still needed to better comprehend the molecular mechanisms involved in GBM tumorigenesis. To this end, the goal of this study was to characterize the chromosomal composition based on copy number alteration (CNA) and transcriptome profile of a newly established glioblastoma cell line, as a strategy to discover potential druggable targets that might prevent GBM development and progression.

## Material and Methods

### Study approval by the Research Ethics Committee and collection of non-neoplastic samples

This study was approved by the Research Ethics Committee of the Instituto Evandro Chagas/IEC/SVS/MS (Process Number 192.336). The use of the sample to establish the AHOL1 cell line was approved by the Research Ethics Committee of the Ophir Loyola Hospital, and a written informed consent was obtained from the patient.

Ten non-neoplastic samples from nervous tissue were obtained from biopsies of patients from Ophir Loyola Hospital, Belém, Brazil. All tissue samples were immediately frozen in liquid nitrogen and stored in DNA/RNA Shield™ (Zymo Research, USA) at -80°C until the extraction stage.

### Materials

Dulbecco's modified Eagle's medium (DMEM), fetal bovine serum (FBS), trypsin/EDTA, penicillin G, and streptomycin were obtained from Gibco (USA) and used to grow GBM cell lines in culture.

### Culture of human glioblastoma cell lines

The main subject of this study was the AHOL1 (Astrocytoma Ophir Loyola Hospital 1) cell line established by our group at the Human Cytogenetics Laboratory, Federal University of Pará (UFPA), from a secondary GBM obtained from the tumor resection of a 43-year-old multiracial male patient treated at the Neurological Clinic of Ophir Loyola Hospital (Brazil) with a histopathologic diagnosis of GBM (grade IV) that evolved from a grade III astrocytoma (3). For comparison to common alterations observed in glioblastoma cell lines, we used three well established human glioblastoma cell lines in our experiments: U-343 MGa cell line kindly provided by the Cytogenetics and Mutagenesis Laboratory, University of São Paulo (Ribeirão Preto, Brazil), derived from a primary GBM from a Caucasian adult patient and obtained from CLS Cell Lines Service (CLS order number 300365), U-87 MG purchased from American Type Culture Collection (catalog number ATCC^®^ HTB-14™), and 1321N1 cell obtained from European Collection of Authenticated Cell Cultures (ECACC, catalog number 86030402).

All cell lines were cultured separately in 25-cm^2^ culture flasks using DMEM supplemented with 10% FBS, 100 U/mL penicillin, and 100 µg/mL streptomycin at 37°C in a humidified 5% CO_2_ atmosphere. The medium was changed every 2-3 days, and cells were sub-cultured when confluency reached 70-80% using 0.25% trypsin at 37°C.

### Nucleic acids extraction

When cells reached total confluence, they were washed with PBS, detached with 0.25% trypsin/EDTA (Gibco™), and suspended in PBS. DNA and RNA were extracted using the Wizard^®^ Genomic DNA Purification kit (Promega Corporation, USA) and SV Total RNA Isolation System (Promega Corporation), respectively, according to the manufacturer’s protocol.

DNA and RNA purity and integrity were assessed on the Agilent 2200 TapeStation (Agilent Technologies, USA) with D1000 ScreenTape (Agilent Technologies) and High Sensitivity RNA ScreenTape (Agilent Technologies) respectively, according to the manufacturer’s protocol. Only samples with DNA Integrity Number (DIN) and RNA integrity (RIN) >7 were used for downstream analyses.

### Karyotype characterization

For cytogenetic analyses, three different passages of the cell line were cultured and blocked by adding 100 µL colcemid (0.0016%). After incubation for one hour, cells were harvested with 0.05% trypsin, incubated with hypotonic solution (0.075 KCl) for about 20 min at 37°C, and fixed with 3:1 methanol/acetic acid. Afterwards, slides were prepared and submitted to standard Giemsa staining and G-banding using trypsin (4). The modal diploid number was defined after analysis of 50 metaphase plates. Chromosomes were arranged and described following the recommendations of the International System for Human Cytogenetic Nomenclature (ISCN).

### Array-based comparative genomic hybridization (aCGH) analysis

*Chromosomal imbalances analysis*. Array-CGH (aCGH) experiments were performed on an Agilent microarray platform (Agilent Technologies) with a SurePrint G3 Cancer CGH+SNP Microarray 4x180K slide (Agilent). Sample preparation, labelling, and microarray hybridization were performed according to the Agilent CGH Enzymatic Protocol version 7.5. Slides were scanned using the Agilent G2565CA scanner. Data were extracted with Feature Extraction software (v9.1 Agilent Technologies) and analyzed with Genomic Work Bench 11.0.1, Agilent Cytogenomics 5.0 and GeneSpring GX 14.5, as described elsewhere (5). The algorithm used was Aberration Detection Method 2 (ADM-2), applying the following filters: threshold=6; minimum number of probes in region=3; and Log2Ratio >0.25 and log2Ratio <-0.25 were defined as copy number gains and losses, respectively. Furthermore, we included the SNP Copy Number (confidence level 0.90), GC correction, diploid peak centralization, and LOH (threshold 6.0) in our analysis.

The ideogram showing the CNAs identified in AHOL1 genome was constructed using the PhenoGram online software (https://ritchielab.org/software/phenogram-downloads). The CNAs information of 1087 cancer cell lines from CellMiner and the Cancer Cell Line Encyclopedia (CCLE), 1987 human GBMs from TCGA database, stored in the cBioPortal for Cancer Genomics (accessed in June 2019) was used to explore the similarities with CNAs of AHOL1 cell line. Additionally, Candidate Cancer Gene Database (CCGD) was used to identify candidate cancer genes from CNAs of AHOL1 genome.

*Gene expression microarray*. Gene expression profiling analysis was performed using the Agilent Oligo Microarray Kit 8×60K according to the Agilent One-Color Microarray-based Gene Expression Analysis Protocol (Agilent Technologies). Data were extracted with Feature Extraction software (v9.1, Agilent Technologies) and analyzed with GeneSpring software GX 14.5 (Agilent Technologies). Raw data were normalized by robust multiarray average (RMA) quantile normalization analysis algorithm with the GeneSpring GX 14.5 software (Agilent Technologies) to generate CEL intensity files and the ratios were log2-transformed for multiple testing. We performed the quality control following diagnostic plots: principal component analysis (PCA), boxplots, Pearson’s correlation, and MvA plots. All gene expression microarrays experiments were performed in triplicate.

Significantly differentially expressed genes (DEGs) were identified by using the mixed model analysis of variance with absolute fold-change values ≥2. To reduce the false discovery rate (FDR), the P value for significant differences was set to less than 0.05.

Gene ontology enrichment analysis was performed using DAVID (Database for Annotation, Visualization and Integration Discovery) bioinformatics tools. Pathway analysis was performed with GeneSpring software GX 14.5 (Agilent Technologies) and the pathways were downloaded from the WikiPathways database (https://www.wikipathways.org/index.php/WikiPathways), BioCyc database (BioCyc.org), and KEGG database (https://www.genome.jp/kegg/pathway.html).

### Integrative analysis of CNAs and DEGs

To identify the significant genes that exhibited CNA and gene expression alterations, we integrated the significant CNAs and DEGs using GeneSpring software GX 14.5 (Agilent Technologies) as described elsewhere (5).

### Search for drugs targeting CNAs

The Drug-Gene Interaction Database (DGIdb; https://www.dgidb.org/) was used to search potential drugs targeting CNAs. This database provides gene druggability information from different databases (such as Therapeutic Target database, DrugBank, Pharmacogenomics Knowledge database, papers, and web resources).

### Reverse transcription qPCR (RT-qPCR)

For the cDNA synthesis, we used the GoScript™ Reverse Transcription System (Promega Corporation) following the manufacturer's instructions. Real time PCR (qPCR) was performed as described by Ferreira et al. (6), using GoTaq^®^ Probe qPCR Master Mix (Promega Corp.). All reactions were carried out in triplicate in 96-well PCR plates, using CFX96 Touch™ Real-Time PCR Detection System (Bio-Rad, USA). Data analysis was performed using the Bio-Rad CFX Manager™ 3.1 software (Bio-Rad). Following the MIQE guidelines, the expression levels were normalized using TBP and GAPDH in non-neoplastic samples. The relative gene expression was calculated using the 2^−ΔΔCT^ formula (P<0.05).

The expression of the genes *ANOS1*, *ETV1*, *XPNPEP2*, and *PCDH11Y* was quantitated using Taqman^®^ gene expression assays (Applied Biosystems, USA) (Table 1).

**Table 1:** Targets and housekeeping genes used in this study.

Official gene symbol^*^	Official full name^*^	Assay ID
GAPDH	Glyceraldehyde-3-phosphate dehydrogenase	Hs02786624_g1
TBP	TATA-box binding protein	Hs00427620_m1
ANOS1	Anosmin 1	Hs01085107_m1
ETV1	ETS variant 1	Hs00951951_m1
XPNPEP2	X-prolyl aminopeptidase 2	Hs00950918_m1
PCDH11Y	Protocadherin 11 Y-linked	Hs06651077_g1

*Official symbols and names of the genes were based on HUGO gene nomenclature committee (HGNC).

## Results

### Cytogenetic characterization

**Figure 1:**
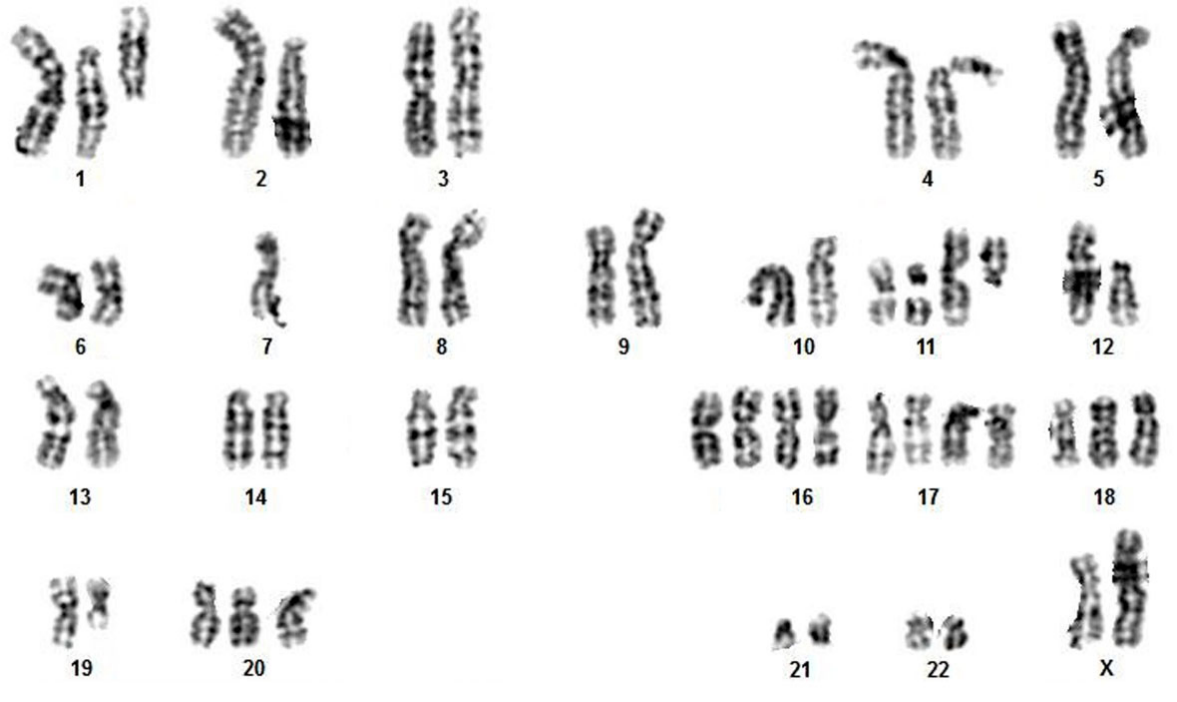
A representative G-banded metaphase of AHOL1 cell line, with 2n=54, XX, +der(1), +der(1), +der(2), del(6q), del(6q), -7, +der(11), +der(11), del (11q), del(12p), +16, +16, +17, +17, +18, del(19q), +der20.

We obtained and analyzed 50 chromosome spreads of the AHOL1 cell line. No normal karyotype was found in any of the metaphases analyzed. All metaphases were hyperdiploid, with chromosome number varying from 2n=51 to 2n=59, with a modal number of 54 and 57, both showing a frequency of 26% each (13/50). G-banding analysis confirmed the occurrence of both numerical and structural rearrangements, with loss/gain of chromosomes or chromosome arms, and fission of some pairs. Most numerical rearrangements involved gain of chromosomes of group E. Despite the fact of AHOL1 has been obtained from a male patient, the chromosome Y was missing in all the metaphases analyzed by G-band. Additionally, most cells gained an extra copy of chromosome X. A representative karyotype is shown in Figure 1. An overall idea of common quantitative alterations were detected by aCGH.

### CNA profiling of AHOL1 cell line

**Figure 2:**
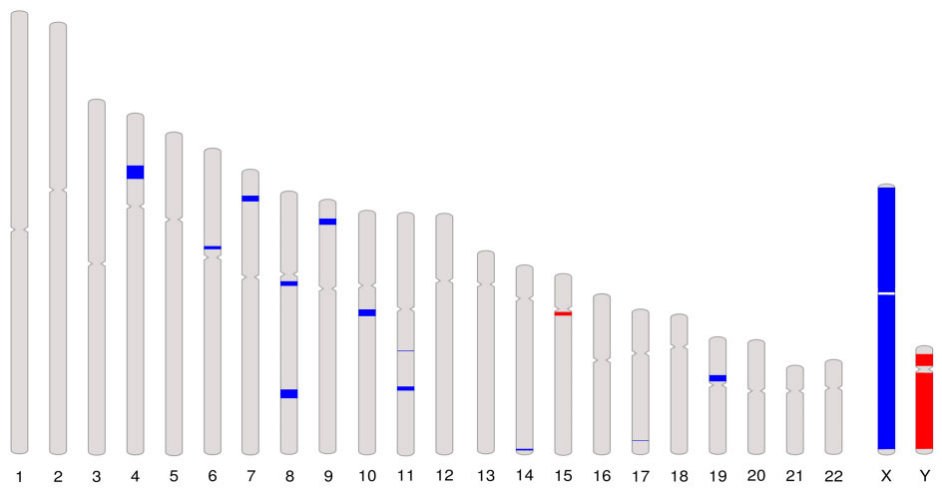
Representative copy number alteration (ideogram) showing gains (blue), and losses (red) in the AHOL1 genome.

A global view of the AHOL1 CNAs composition was generated using the a-CGH results. A total of 19 CNAs (17 gains and 2 losses) were identified, ranging from 0.28 Mb to 93 Mb. A full list of the CNAs and their corresponding chromosome localization, cytobands, type of alteration, P value, and genes are provided in Supplementary Table S1. Copy number gains were located at chromosomes 4, 6, 7, 8, 9, 10, 11, 14, 17, and 19, while losses were at chromosome 15 (Figure 2; Supplementary Table S1). Whole chromosome gains and losses were observed at chromosomes X and Y, respectively.

The highest number of gains was found at chr X at q11.1-q28 (93,148.679 kb - with 3016 segments), p22.33-p11.1 (55,799.123 kb - with 1745 segments), and q21.1-q21.33 (13,545,889 kb) positions. The second highest number of gains was documented at chromosome 4 (cytoband p15.1-p14) corresponding to 7,800 kb, followed by chromosome 8 (cytoband q23.2-q23.3) corresponding to 5,104 kb. Additionally, we identified that the highest frequency of losses occurred on the Y chromosome at q11.21-q12 (45,327.039 kb) and p11.31-p11.2 (7,119.122 kb) positions (Figure 2).

We further explored whether AHOL1 CNAs were recurrent in cancer cell lines from CellMiner and CCLE databases (N=1087 cell lines) (≥10% of frequency). Indeed, *in silico* analysis revealed that thirty-one genes were covered by CNAs in most cancer cell lines (Supplementary Table S2). In addition, by performing Ingenuity Pathway Analysis (IPA), a total of 240 cancer-related genes, 15 of them exclusively related with brain cancer (Supplementary Table S3) were revealed. Also, we analyzed whether CNAs detected in AHOL1 were frequently found across primary GBM tumors from the TCGA database. Interestingly, the vast majority of genes covered by CNAs were commonly altered across several primary GBM tumors.

We also conducted an analysis to detect the main altered pathways affected by CNAs in the AHOL1 genome. Amplifications affected 60 pathways, such as putrescine degradation III, melatonin degradation II, and leucine degradation pathways, while losses had no impact in any pathway (Supplementary Table S4).

### AHOL1 is genomically similar to other human GBM cell lines

**Figure 3:**
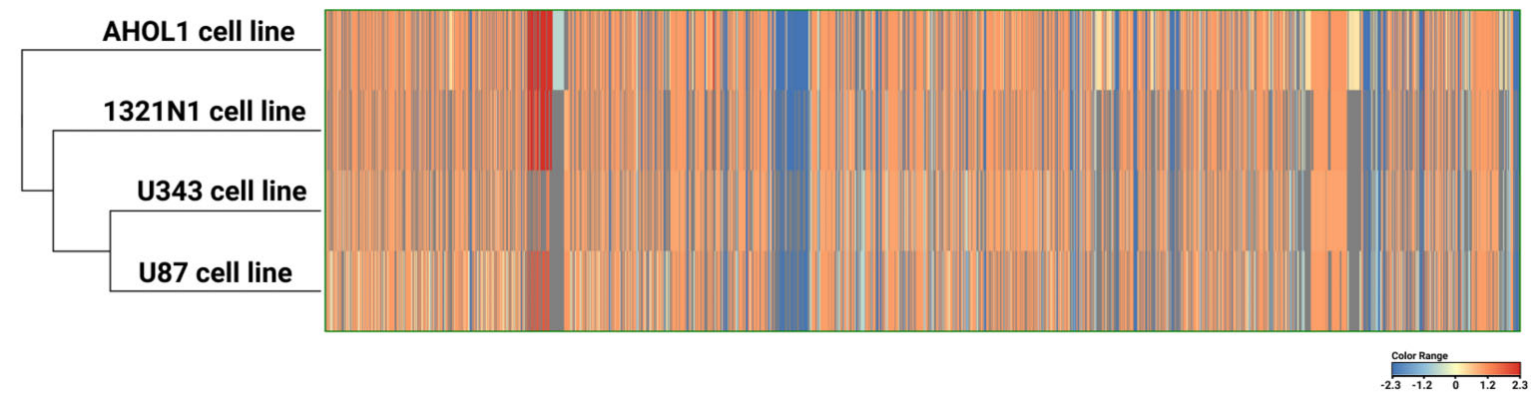
Heatmap of all copy number alterations present in each cell line (AHOL1, 1321N1, U343, and U87).

Considering that the AHOL1 cell line was established from a GBM patient, we expected it to share common CNAs with commercial GBM cell lines (U87MG, U343, and 1321N1). As shown in Figure 3, our results indicated the existence of a common genomic signature between AHOL1 and commercial GBM cell lines (1321N1, U343, and U87), thus confirming its GBM identity.

### Drugs targeting CNAs

Potential drugs targeting CNAs were investigated using the DGIdb database. Nine druggable genes were identified, including *HCRTR2* (hypocretin receptor 2), *ETV1* (ETS variant 1), PTPRD (protein tyrosine phosphatase, receptor type D), *PRKX* (protein kinase X-linked), *STS* (steroid sulfatase), *RPS6KA6* (ribosomal protein S6 kinase A6), *ZFY* (zinc finger protein Y-linked), *USP9Y* (ubiquitin specific peptidase 9 Y-linked), and *KDM5D* (lysine demethylase 5D) (Table 2).

**Table 2:** Druggable genes of the AHOL1 cell line. Information of each gene regarding their chromosomal location, cytoband, size (kb), type of alteration found (gain or loss), drugs, and the type of interaction of each drug is provided below.

Chromosome	Cytoband	Size (Kb)	Variant type	Gene	Drug	Interaction type
6	p12.1	1,752	Gain	HCRTR2	Suvorexant	Antagonist
					SB-649868	Antagonist
					Lemborexant	Antagonist
					Almorexant	n/a
7	p21.3 - p21.2	3,393	Gain	ETV1	Trametinib	n/a
10	q21.1	3,864	Gain	PTPRD	Cucurbitacin	n/a
					Teprotumumab	n/a
					Cixutumumab	n/a
X	p22.33 - p11.1	55,799	Gain	PRKX	GSK-690693	Inhibitor
			Gain	STS	Estrone	n/a
					Danazol	n/a
					Triptorelin	n/a
					Penicillamine	n/a
					Progesterone	n/a
					STS	Inhibitor
X	q21.1 - q21.33	13,545	Gain	RPS6KA6	AT-9283	Inhibitor
					Chembl573107	Inhibitor
Y	p11.31 - p11.2	7,119	Loss	ZFY	Chembl383208	
Y	q11.21 - q12	45,327	Loss	USP9Y	Testosterone	n/a
				KDM5D	Ascorbate	n/a

n/a: not available.

### Transcriptome characterization of AHOL1 cell line

In total, we identified 1,837 DEGs. Among these, 713 genes were upregulated, whereas 1,124 genes were downregulated (FC ≥2 and P<0.05) (Table 3). A full list of differentially expressed genes and their corresponding fold changes in expression and P values are provided in Supplementary Table S5. Ret Finger Protein-like 4A-like 1 (*RFPL4AL1*) was the most upregulated (FC: 55.87), and lincRNA *lnc-CHIC1-2:1* the most downregulated mRNA (FC: -65.82) (Table 3).

**Table 3:** Characteristics of the top 20 differentially expressed mRNAs in AHOL1 cell line sorted by fold change (fold change ≥2 and P<0.05).

	Gene symbol	Chromosome	Description	Fold change
Upregulated	RFPL4AL1	chr19	Ret Finger Protein-like 4A-like 1	55.87
	lnc-WDR5-2	chr9	lincRNA	27.04
	ERICH1-AS1	chr8	lncRNA	26.70
	LRRN4	chr7	Leucine Rich Repeat Neuronal 4	21.44
	CMTR1	chr6	Cap Methyltransferase 1	21.38
	XLOC_l2_012743	chr6	lncRNA	11.53
	LINC01297	chr14	lincRNA	10.86
	KDM4E	chr11	Lysine demethylase 4E	9.08
	lnc-RBPJ-1: 1	chr4	lincRNA	8.59
	lnc-FBXO25-5: 4	chr8	lincRNA	6.21
Downregulated	lnc-CHIC1-2: 1	chrX	lincRNA	-65.82
	SHANK3	chr22	SH3 and multiple ankyrin repeat domains 3	-36.71
	SNORD114-3	chr14	Small nucleolar RNA, C/D box 114-3	-18.68
	LOC403323	chr9	Uncharacterized LOC403323	-12.19
	ZFP57	chr6	ZFP57 zinc finger protein	-10.78
	CYP11A1	chr15	Cytochrome P450 family 11 subfamily A member 1	-9.88
	DSP	chr6	Desmoplakin	-9.16
	PLVAP	chr19	Plasmalemma vesicle associated protein	-8.89
	C4BPA	chr1	Complement component 4 binding protein alpha	-8.72
	lnc-ZNF100-2	chr19	lncRNA	-7.92

**Figure 4:**
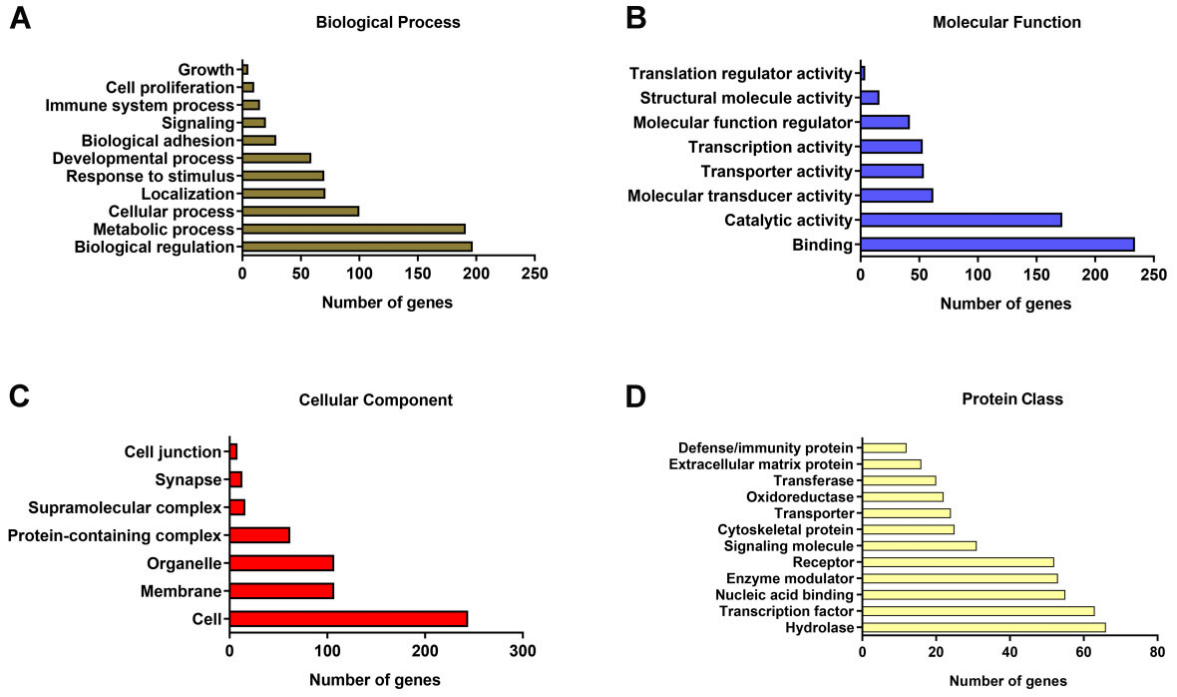
Gene Ontology (GO) functional annotations for differentially expressed genes (DEGs) of the AHOL1 cell line. Bar graphs show four GO categories: (**A**) Biological processes, (**B**) Molecular functions, (**C**) Cellular components, and (**D**) Protein classes. The X-axis represents the number of DEGs and the Y-axis shows the GO terms of each category (fold change ≥2 and P<0.05).

To better understand the biological processes associated with DEGs, Gene Ontology (GO) analysis was conducted. The majority of DEGs were distributed into four GO categories: biological process, molecular function, cellular component, and protein class (Figure 4). A full list of GO terms is provided in Supplementary Table S6.

In the biological process category, the most enriched terms were related to biological regulation and metabolic process (Figure 4A). Binding and catalytic activity mostly accounted for terms related to the molecular function category (Figure 4B). Within the cellular component category, the GO term with the highest level of significance was cell, followed by membrane and organelle (Figure 4C). Finally, in the protein class category, the terms hydrolase and transcription factor exhibited the highest significance (Figure 4D).

Pathway analysis was performed to investigate the biological significance of these DEGs. Thirteen pathways were significantly affected in the AHOL1 cell line, such as mevalonate, IL-1, glycogenolysis, and mRNA capping pathways in cancer (Table 4).

**Table 4:** Pathway analysis of differentially expressed genes from AHOL1 cell line.

Pathways	P value
**Upregulated**	
Trans, trans-farnesyl diphosphate biosynthesis	0.0262
Superpathway of geranylgeranyldiphosphate biosynthesis I (via mevalonate)	0.0391
Mevalonate pathway	0.0391
IL1	0.0397
4-hydroxy-2-nonenal detoxification	0.0414
Glycogenolysis	0.0311
mRNA capping	0.0417
**Downregulated**	
Pathways in cancer	0.0091
C20 prostanoid biosynthesis	0.0169
Plasmalogen degradation	0.0229
Rapoport-Luebering glycolytic shunt	0.0229
Pyridoxal 5'-phosphate salvage	0.0229
Choline degradation	0.0229

Pathways were selected according to the P value (fold change ≥2 and P<0.05).

### AHOL1 cell line shares several DEGs with other commercial GBM cell lines

To determine the number of genes shared between AHOL1 and the commercial GBM cell lines, Venn diagrams were created. Our results showed that AHOL1 shared several transcripts with the commercial GBM cell lines.

**Figure 5:**
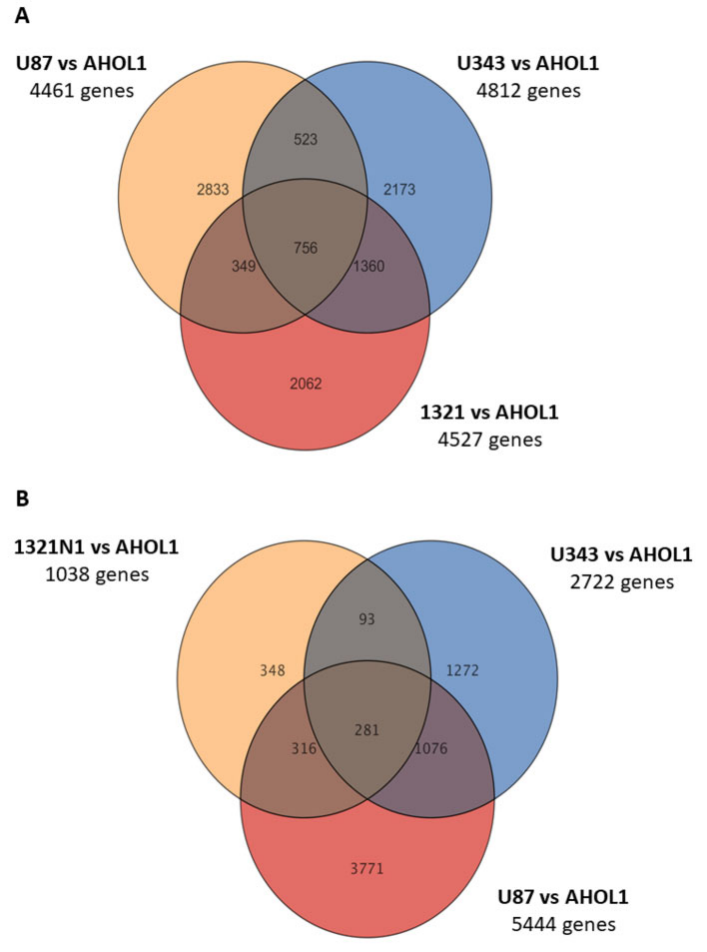
Venn diagram showing the number of genes upregulated (**A**) and downregulated (**B**) in all glioblastoma cell lines.

Analysis of transcriptomes highlighted that the AHOL1 cell line has several changes common to the different GBM commercial cell lines. All cell lines had shared 756 upregulated and 281 downregulated genes (Figure 5).

### Integrative analysis of CNA and gene expression profiling

To explore and compare how CNAs affect AHOL1 transcriptional program, we performed an integrative analysis of CNAs with DEGs. Most of DEGs showed no positive correlation with CNAs. Only 57 genes displayed significant CNA-DEGs correlation in the AHOL1 cell line. A full list of these overlapping genes is found in Supplementary Table S7. Twenty-four genes showed a positive association, while 33 genes presented an inverse association (Table 5).

**Table 5:** Overlapping genes identified after integration of copy number alteration (CNAs) and gene expression data of AHOL1 cell line.

CNA	Gene expression	Overlapping genes
Gain	Upregulated	13
Gain	Downregulated	32
Loss	Upregulated	11
Loss	Downregulated	1
Total		57

**Figure 6:**
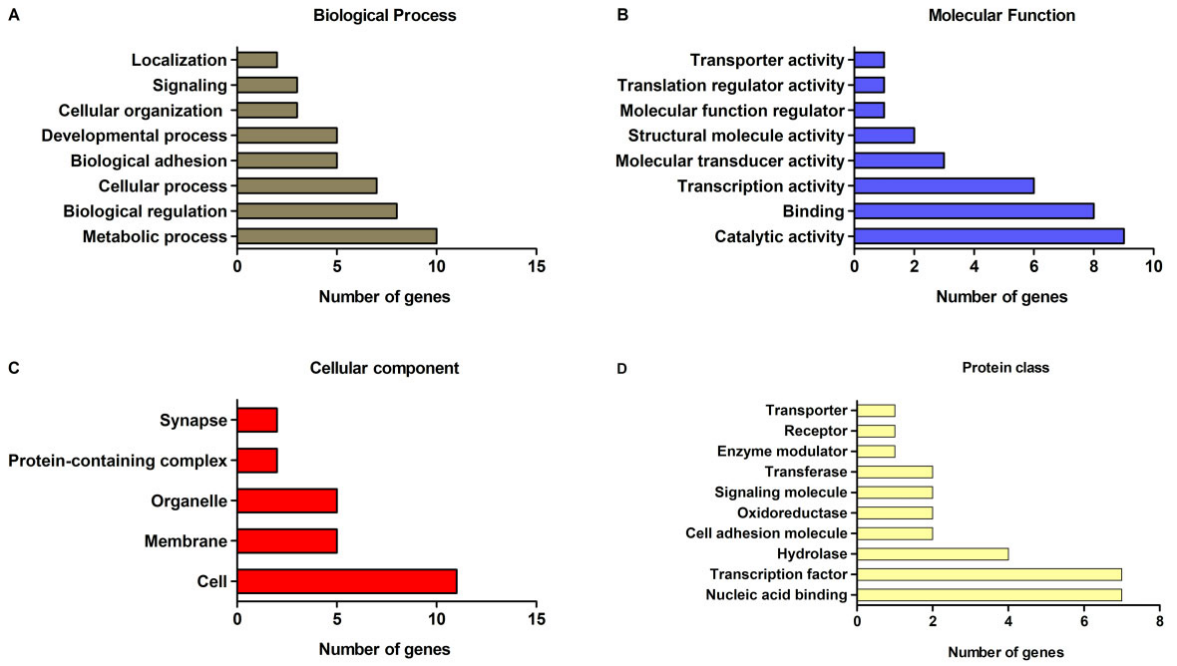
Functional classification of integrated differentially expressed genes (DEGs) copy number alterations (CNAs) data from AHOL1 cell line using Gene Ontology (GO) analysis. Bar graphs show four independent GO information categories: (**A**) Biological processes, (**B**) Molecular functions, (**C**) Cellular components, and (**D**) Protein classes. The Y-axis represents the number of genes of integrated DEGs-CNAs data and the X-axis shows the GO terms for each category (P<0.05).

To further understand the biological function of these 57 overlapping genes, they were submitted to functional annotation and classification analysis. Most of these genes were enriched for metabolic processes, biological regulation, and cellular processes in the biological category (Figure 6A). For the molecular function category, the GO terms with the highest levels were catalytic activity, binding, and transcription activity (Figure 6B). Within the cellular component category, cell was the highest term, followed by membrane and organelle terms (Figure 6C). Of note, in the protein class category, the terms nucleic acid binding and transcription factor exhibited the highest frequencies (Figure 6D).

Next, we performed separately GO functional annotation of each group shown in Table 5. As shown in Supplementary Table S8, most genes covered by gains whose expression was upregulated mainly affected the nucleus (Cellular Component), provoking changes in the DNA-binding transcription factor (TF) activity (Molecular Function) of the helix-turn-helix and zinc finger TFs (Protein Class), which consequently had an effect on the cell development and cell differentiation (Biological process).

Genes covered by gains and whose expression was downregulated mostly affected the membranes (Cellular Component), especially disturbing the hydrolase activity (Molecular Function), modifying the cell communication, signal transduction, cellular response to stimulus, and cellular metabolic process (Biological Process). Furthermore, genes covered by losses and whose expression was upregulated essentially affected the cytoplasm (Cellular Component), altering the RNA binding (Molecular Function) of the ribosomal proteins and translation factors (Protein Class) and influencing the translational elongation and termination (Biological Process). Finally, *PCDH11Y* loss and down-regulation affected plasma membrane, by altering the cell adhesion and inducing changes in the calcium ion binding (Supplementary Table S8)

We also conducted a global analysis with all 57 genes from the DEGs-CNAs dataset in order to detect the main altered pathways from AHOL1. Signaling pathways via Receptor-type tyrosine protein phosphatases, Cadherin, Wnt, FGFR1, and Akt, as well as metabolism of proteins were statistically significant.

### qRT-PCR validation of the microarray results

**Figure 7:**
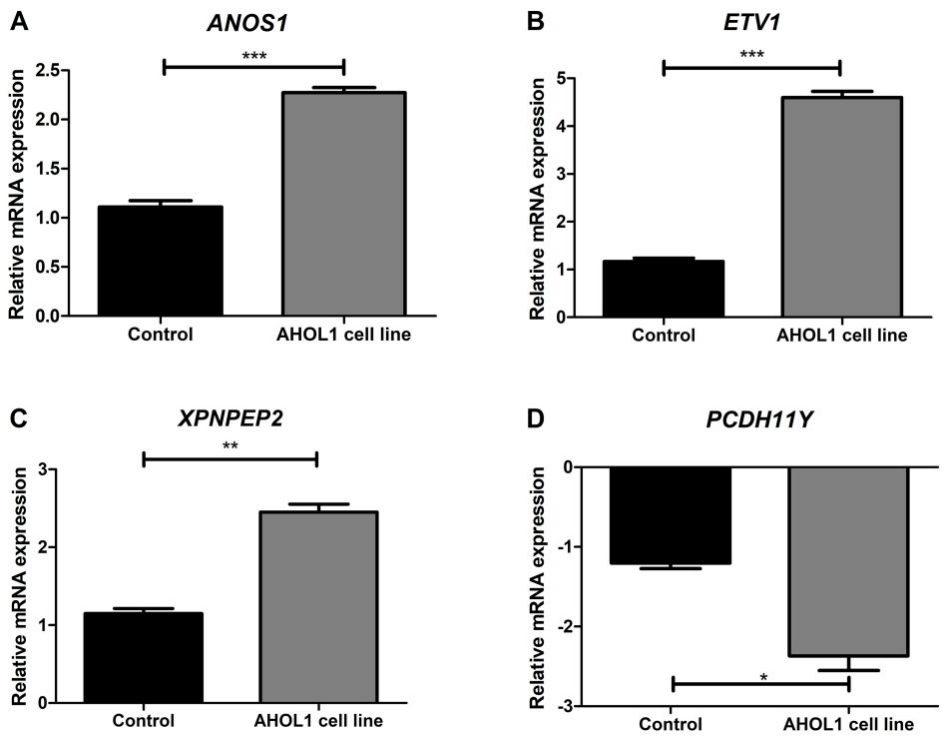
Validation of the microarray data by qRT-PCR. qRT-PCR was performed for *ANOS1* (**A**), *ETV1* (**B**), *XPNPEP2* (**C**), and *PCDH11Y* (**D**) genes. Data are reported as fold change in mRNA expression and compared with a tissue fragment from normal brain as a control. Each bar represents means±SD values for each gene with three technical replicates. Statistical significance was determined using Student's *t*-test: *P<0.05; **P<0.01; ***P<0.001.

To validate the DEGs-CNAs dataset generated by microarray, we selected four genes related to cancer. Three of them (*ANOS1*, *ETV1*, and *XPNPEP2*) were upregulated via copy number gain and one gene (*PCDH11Y*) was downregulated via copy number loss. Transcription levels of selected genes are shown in Figure 7.

Gene expression showed that *ANOS1*, *ETV1*, and *XPNPEP2* were upregulated in AHOL1 relative to normal brain tissue fragments (2-fold; >4 fold; 2.7-fold, respectively), which is in line with the microarray data (Figure 7 and Supplementary Table S5). Of note, *PCDH11Y* was 2.3-fold downregulated in AHOL1 relative to normal brain tissues, in agreement with the microarray data (Figure 7D; Supplementary Table S5).

## Discussion

Recent data provided by The Cancer Genome Atlas (TCGA) consortium have shown great genetic and epigenetic diversity among GBM tumors (1). The elucidation of the biological mechanisms and the complexity behind this diversity is a central challenge for achieving precision medicine in GBM subtypes. For this reason, the availability of a large number of molecularly well-characterized GBM cell lines may have a high impact on understanding the complex biology of this tumor, thus contributing to identification of new therapeutic targets.

As an attempt to better understand the complexity of the GBM biology and the phenomena that pervade the aspects of its intricate mechanisms, we provide the first comprehensive cytogenomic profile of the human glioblastoma cell line AHOL1. This cell line showed a hyperdiploid karyotype, and clearly exhibited recurrent CNAs consistent with those observed in primary GBM tumors from TCGA database, CellMiner, and Cancer Cell Line Encyclopedia, as well as the commercial GBM cell lines (1321N1, U343, and U87) used in this study and other reports (7,8). Among these, the most prominent CNAs were documented at X- (gain of entire chromosome) and Y-chromosomes (loss of entire chromosome), in line with previous cytogenetics and molecular reports for GBM tumors (9).

Chromosomal rearrangements are common in cancer cells, and it is proposed that they influence cancer development, progression, metastasis, and drug resistance due to possible alteration in gene expression, not only by gain/loss, but also by translocations. Thus, a better understanding of cancer-causing chromosomal rearrangements may enable the development of novel anticancer protocols (10). In this sense, although many functional aspects of CNAs at chr X and chr Y have not yet been extensively studied in cancer, a preliminary study suggested that high expression of *SPIN4* and *ASB12* was correlated with amplification at Xq11 in GBM (11), thus affecting several important biological processes, such as mitosis, Wnt signaling pathway, H3K4me3, and post-translational protein modifications. Recent reports also support that the X chromosome gain plays a prominent role in the neoplastic transformation of breast cancer (12), chronic neutrophilic leukemia (13), non-Hodgkin lymphoma (14), renal cell carcinoma (15), and prostate cancer (16).

Massive amounts of cancer genomics data generated from next-generation sequencing have motivated investigators to develop novel computational approaches for the identification of new druggable genes, which can be used as therapeutic targets in precision cancer medicine. Our AHOL1 genomic data showed that nine genes (*HCRTR2*, *ETV1*, *PTPRD*, *PRKX*, *STS*, *RPS6KA6*, *ZFY*, *USP9Y*, and *KDM5D*) are known as potential druggable anticancer targets, and some of them could be druggable vulnerabilities in some subtypes of GBM (17). Together, these studies have highlighted the great potential of AHOL1 cell line for *in vitro* studies.

The association between chromosome rearrangements, including CNAs, and gene expression profiling suggested the identified alterations could contribute to the expression of some but not all genes. In some cases, expression changes were inconsistent with the CNAs. This might be influenced by other factors that contribute to gene expression variation, such as epigenetics changes, non-coding RNA regulation, gene mutation, and altered expression of TFs (18). This apparent discrepancy between copy number status and gene expression has also been observed in other cancers (19).

In this study, pathway enrichment of CNA-driven DEGs indicated significant changes in six pathways, where the majority of genes were prominently involved in the signaling by tyrosine phosphorylation. Tyrosine phosphorylation plays an important role in regulating cellular function and is a central feature in signaling cascades involved in oncogenesis. This process is coordinately controlled by protein tyrosine phosphatase (PTPs) and protein tyrosine kinases (PTKs), which are altered in a variety of human cancers, including GBMs (20). Among the tyrosine phosphatases, the tumor suppressor *PTPRD* is one of those often inactivated by deletion (>50% of cases), whose loss of expression promotes gliomagenesis through aberrant STAT3 activation (21) and is related with poor prognosis in GBM patients. Our observations, therefore, suggested that this gene was amplified and downregulated in AHOL1. Perhaps, this gene might be epigenetically silenced in AHOL1, once GBMs that do not harbor loss of *PTPRD* have this gene inactivated by its promoter hypermethylation (22).

Assuming that CNA-DEG integrated data have been shown to be an efficient approach to identify genes covered by altered CNAs that directly change their expression levels, not all genes of our integrated dataset were cancer-related. In order to explore only genes related to cancer, we selected four genes, including three that were upregulated via copy number gain (*ANOS1*, *ETV1*, and *XPNPEP2*) and one gene (*PCDH11Y*) that was downregulated via copy number loss.

*ANOS1*, also known as *KAL1*, encodes anosmin-1, an extracellular matrix (ECM)-associated protein that plays essential roles in neural cell adhesion and axonal migration. The upregulation of this gene in the AHOL1 cell line was consistent with the results from Choy et al. (23) for GBM and low-grade astrocytic tumors, as they found that anosmin-1 enhanced cell motility and proliferation in GBM cell lines. The overexpression of *ANOS1* is also related to development and metastasis of colorectal cancer and its expression is closely related to the overall survival rate of patients (24).

Previous studies have shown that the oncogene *ETV1* (ETS variant 1 - member of the ETS family of transcription factors) contributes to neoplastic transformation of prostate cancer (25), breast cancer (26), esophageal adenocarcinoma (27), gastrointestinal stromal tumors (28), cranial germinomas (29), gastric cancer (30), melanoma brain metastases (31), and melanoma (32). In GBMs, high expression of *ETV1* may be a central downstream effector of chromosome 7 gain (33). Additionally, *ETV1* is likely to be methylated in CIC wild-type, IDH-mutated, 1p/19q-codeleted gliomas (34), and fused with DGKB (35) in pediatric high‐grade gliomas, acting as an oncogenic driver. In line with these results, we here demonstrated that the up-regulation of *ETV1* was due to a chr7 gain in AHOL1 genome. Thus, the high prevalence of upregulated *ETV1* together with chromosome 7p gain in GBMs may be new targets for efficacious therapies to improve outcomes of patients.

*XPNPEP2* is a membrane-bound aminopeptidase P member of the ‘pita bread fold' family, which catalyzes the removal of the penultimate prolyl residue from N-termini of peptides. Although it is known that this aminopeptidase activates growth factors, hormones, coagulants, toxins, cytokines, and neurotransmitters, the role of *XPNPEP2* in cancer is still unknown. It is known that *XPNPEP2* is overexpressed in cervical cancer, promoting cell invasion and migration without affecting cell proliferation and apoptosis (36). Our gene expression results corroborated these findings and point out that *XPNPEP2* can be upregulated in GBM.

Protocadherins constitute the largest subfamily of cadherins in the genome. Protocadherins genes are predominantly expressed in the nervous system, acting in crucial functions associated with formation, maintenance, and integrity of the neural circuit (37). Recently, they have been in the spotlight for their roles in cancer (38). Protocadherin-11Y (also named as *PCDH11Y*) is a proto-oncogene candidate exclusively found in man and its transcription occurs mainly in the brain (37). This gene is upregulated in prostate carcinoma (39), inducing neuroendocrine transdifferentiation through activation of the wnt signaling (40). To the best of our knowledge, this is the first study showing that *PCDH11Y* was downregulated in GBM, contradicting the results found elsewhere (39).

In summary, we described for the first time the genome and transcriptome of the new human cell line AHOL1, established from a GBM patient. Here, we revealed that this cell line harbored a genomic alteration spectrum similar to what is observed in commercial GBM cell lines (U87MG, U343, and 1321N1) and GBMs from the TCGA database, and some of these CNAs can be targeted by drugs, suggesting that this new cell line is a suitable model system for understanding the molecular characteristics of human GBM tumorigenesis.

## Supplementary Material

Click here to view [zip].
